# Limitation of room temperature phosphorescence efficiency in metal organic frameworks due to triplet-triplet annihilation

**DOI:** 10.3389/fchem.2022.1010857

**Published:** 2022-10-31

**Authors:** Tonghan Zhao, Dmitry Busko, Bryce S. Richards, Ian A. Howard

**Affiliations:** ^1^ Institute of Microstructure Technology, Karlsruhe Institute of Technology, Karlsruhe, Germany; ^2^ CAS Key Laboratory of Nanosystem and Hierarchical Fabrication, National Center for Nanoscience and Technology (NCNST), Beijing, China; ^3^ Light Technology Institute, Karlsruhe Institute of Technology, Karlsruhe, Germany

**Keywords:** MOF (metal–organic framework), triplet-triplet annihilation, quantum yield (QY), photoluinescence, photophysics

## Abstract

The effect of triplet-triplet annihilation (TTA) on the room-temperature phosphorescence (RTP) in metal-organic frameworks (MOFs) is studied in benchmark RTP MOFs based on Zn metal centers and isophthalic or terephthalic acid linkers (ZnIPA and ZnTPA). The ratio of RTP to singlet fluorescence is observed to decrease with increasing excitation power density. Explicitly, in ZnIPA the ratio of the RTP to fluorescence is 0.58 at 1.04 mW cm^−2^, but only 0.42 at (the still modest) 52.6 mW cm^−2^. The decrease in ratio is due to the reduction of RTP efficiency at higher excitation due to TTA. The density of triplet states increases at higher excitation power densities, allowing triplets to diffuse far enough during their long lifetime to meet another triplet and annihilate. On the other hand, the shorter-lived singlet species can never meet an annihilate. Therefore, the singlet fluorescence scales linearly with excitation power density whereas the RTP scales sub-linearly. Equivalently, the efficiency of fluorescence is unaffected by excitation power density but the efficiency of RTP is significantly reduced at higher excitation power density due to TTA. Interestingly, in time-resolved measurements, the fraction of fast decay increases but the lifetime of long tail of the RTP remains unaffected by excitation power density. This may be due to the confinement of triplets to individual grains, leading decay to be faster until there is only one triplet per grain left. Subsequently, the remaining “lone triplets” decay with the unchanging rate expressed by the long tail. These results increase the understanding of RTP in MOFs by explicitly showing the importance of TTA in determining the (excitation power density dependent) efficiency of RTP. Also, for applications in optical sensing, these results suggest that a method based on long tail lifetime of the RTP is preferable to a ratiometric approach as the former will not be affected by variation in excitation power density whereas the latter will be.

## Introduction

Long-persistent luminescent (LPL) materials have attracted broad interest in the fields of sensors (Zhou et al., 2019; Katumo et al., 2020), information encryption (Liu et al., 2021), optical imaging (Zhen et al., 2017), and signage (Peng et al., 2021; Liu et al., 2022a). Up to now, most of LPL materials are based on inorganic hosts doped with lanthanide ions (Carvalho et al., 2018; Katumo et al., 2021; Ou et al., 2021). In recent years, the research on organic materials with room-temperature phosphorescence (RTP) been attracting attention due to their large extinction coefficient and more facile integration into optoelectronic devices (Kabe and Adachi, 2017; Kenry et al., 2019; Zhao et al., 2020). The design of organic RTP-active materials is an active field of research with many open aspects including metal-free organic chromophores (Ma et al., 2019), carbon dots (Joseph and Anappara, 2017; Peng et al., 2021), and intermolecular charge-transfer excited states (Zhang et al., 2009). The rigidification of chromophores within metal-organic frameworks (MOFs) have also been revealed to lead to RTP (Mieno et al., 2016; Yang and Yan, 2016; Yang et al., 2019a; Wang et al., 2019).

**FIGURE 1:**
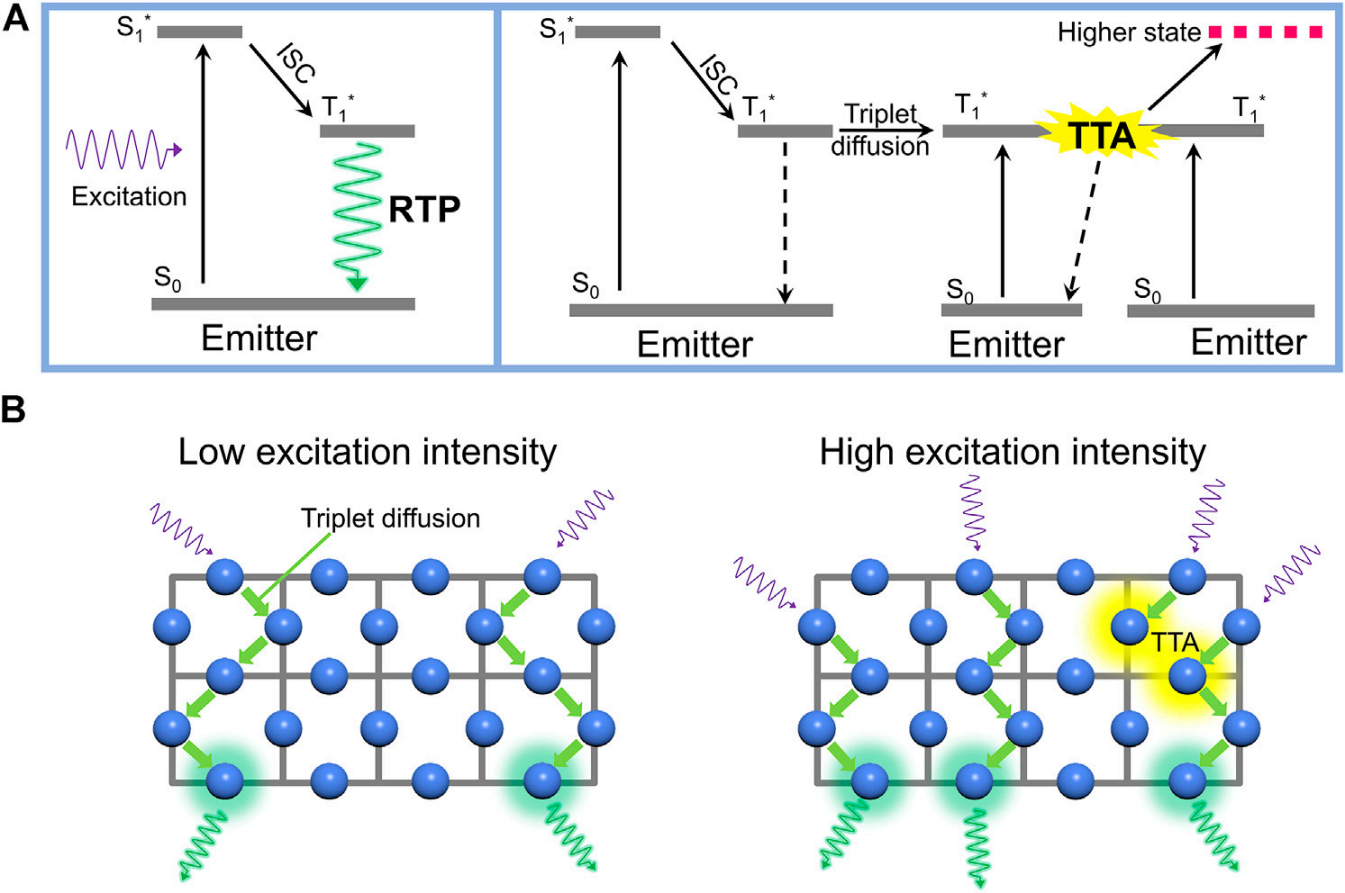
**(A)** After excitation, the room-temperature phosphorescence (RTP) can be generated *via* intersystem crossing (ISC) from excited singlet state to triplet state, afterward the radiative transition of triplet state in organic emitter (left section). Additionally, the triplet excitons also can diffuse to other molecules. When two triplets collide, the triplet-triplet annihilation (TTA) occurs, causing a non-radiative transition of one triplet, the other one gets to a high-level excited state (right section). In this case, the total quantum yield of RTP decreases because of the consumption of triplets *via* TTA **(B)** Sketch of the effect of TTA in RTP materials by comparing the corresponding quantum yields under low excitation intensity (left section) and high excitation intensity (right section).

Usually, photon emission from organic chromophores originates from the singlet excited state radiatively returning to the singlet ground state resulting in fluorescence. This allowed process usually occurs with an inverse rate typically in the range of several nanoseconds. Spin-orbit coupling can induce intersystem crossing (ISC) to populate the triplet excited state from the singlet excited state. The radiative coupling from the triplet excited state is extremely low, with an inverse rate usually on the order of seconds (in the absence of heavy atoms). Normally, the excited triplet state returns to the ground state through a non-radiative process, releasing energy into molecular vibrations. Phosphorescence–the radiative transition from the excited triplet state to the singlet ground state–is usually observed only at low temperatures when the non-radiative rates are suppressed. However, if the non-radiative rate is sufficiently suppressed at room temperature, then RTP can be observed (see left section of Figure 1A). The decay of this emission is typically on the time scale of hundreds of milliseconds or even extending to several seconds. In addition, most RTP-active systems are solid-state (Gao et al., 2021; Ma et al., 2022; Zhou B et al., 2022), which means the organic molecules are tightly stacked together. Therefore, the long-lived triplet excitons and short distance between chromophores should also present an opportunity for triplet diffusion. As sketched in the right section of Figure 1A, if two diffusing triplets collide, triplet-triplet annihilation (TTA) can occur whereby one triplet is promoted to a higher state by the simultaneous relaxation of the second triplet to the ground state. According to the statistical combination of the spin of the encountering triplets, both higher-level excited singlet or triplet states can be generated (Cheng et al., 2010; Hoseinkhani et al., 2015). However, at least one triplet is consumed *via* a non-radiative transition *via* the TTA process instead of creating phosphorescence. Consequently, the absolute quantum yield of RTP is suppressed with the onset of TTA.

In this work, we use two kinds of phosphorescent MOFs as models to verify our hypothesis that the TTA process can limit the emission efficiency of RTP. Luminescent MOFs have received widespread attention owing to their potential application in chemical sensors (Cui et al., 2012a; Hu et al., 2014; Yang et al., 2019b), circularly polarized luminescence (Zhao et al., 2021; Kazem-Rostami et al., 2022; Zhang et al., 2022), biological imaging (Kundu et al., 2016; Lu et al., 2018), and optoelectronic devices (Cui et al., 2012b; Cui et al., 2015; Qin et al., 2021; Qin et al., 2022). In terms of fabricating RTP-active materials, MOFs also exhibited enormous potentials thanks to their long-range ordered structure, the restriction of molecular conformations, and the potential for spin-orbital coupling between metal ions and ligands. RTP-active MOFs with long-lived triplet lifetimes are under active development (Yang and Yan, 2016; Liu et al., 2022b; Zhou Q et al., 2022). Due to the efficient RTP emission and facile synthesis process, isophthalic acid (IPA)- and terephthalic acid (TPA)-based zinc MOFs are used in this work. However, to investigate the influence of TTA on RTP performance, we focus our attention on excitation power dependence of RTP quantum yields. As shown schematically in Figure 1B, upon low-power photoexcitation, discretely distributed triplet excitons produce RTP without collision. The overall efficiency of the RTP process depends on the efficiencies of two events: 1) efficiency of ISC from the singlet; and 2) competition between radiative and non-radiative rate of the triplet. However, under higher-power excitation, the triplet exciton population in the material increases significantly. As the triplets diffuse through the MOF (Park et al., 2018; Adams et al., 2019), an encounter of two triplet excited states can generate a non-radiative decay leading to the loss of at least one triplet state *via* the TTA process. If a singlet state is formed, both triplets in the encounter will be lost. Whereas if an excited triplet state is formed, then only one triplet from the encounter pair is lost. In any case, TTA will cause the RTP emission to scale sub-linearly with increasing excitation power. Given the much shorter lifetime of the singlet, the probability of a singlet undergoing annihilation during its lifetime is much smaller. Therefore, although some singlet-triplet annihilation could decrease the efficiency of singlet emission at high excitation fluences, the RTP will become less significant relative to fluorescence if the long-lived triplets are mobile. In the following we study the change in the ratio of singlet fluorescence to RTP as a function of excitation power density to unambiguously show that TTA plays a role in limiting the quantum yield of triplets in RTP MOFs.

## Results and discussion

### Estimation of QY_RTP_

**FIGURE 2:**
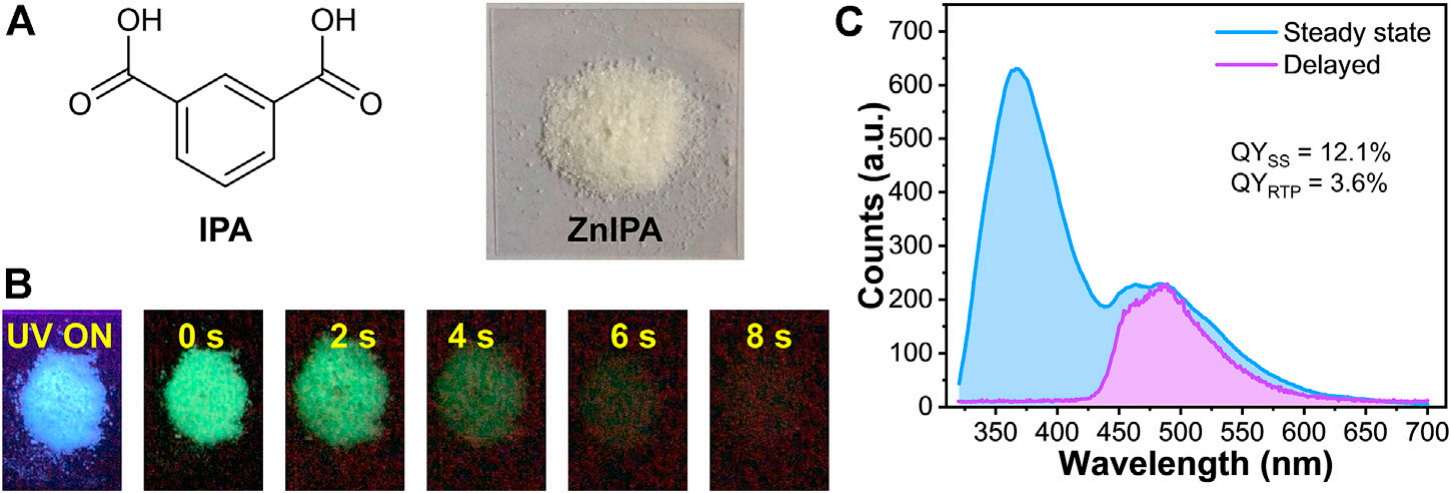
**(A)** Chemical structure of isophthalic acid (IPA) and photograph of ZnIPA MOF **(B)** Photographs of ZnIPA under and after excitation of UV (365 nm) light **(C)** Steady-state photoluminescence (PL) and delayed RTP spectra of ZnIPA. The blue- and purple-colored fillings represent the integrating areas of steady-state PL and delayed RTP, respectively. *λ*_ex_ = 300 nm. For steady-state PL and QY_SS_, the excitation power is 52.6 mW cm^−2^.

Isophthalic acid (IPA, Figure 2A) is one of the popular dicarboxylic linkers in the MOF family because of its diversified coordination modes (Pan et al., 2019). Upon coordination with Zn^2+^ ions *via* a microwave-assisted method (Klinowski et al., 2011), the white powder of ZnIPA MOF was obtained. Under Mercury lamp (365 nm) irradiation, blue emission can be observed from the powder. After the removal of the ultraviolet (UV) excitation source, the ZnIPA exhibited a strong bluish green RTP (Figure 2B). Powder X-ray diffraction (PXRD) measurements confirmed a high purity and crystallinity of ZnIPA (Supplementary Figure S1). The formation of ZnIPA was also confirmed by Fourier-transform infrared (FTIR) spectroscopy. As show in Supplementary Figure S2, after reaction of the IPA with Zn(NO_3_)_2_⋅6H_2_O, the stretching vibration of −OH (−COOH) around 2,800 cm^−1^ disappeared, the vibration peaks at 1,685 (C=O) and 1,266 (C−O) decreased dramatically. These observations confirmed that the coordination of IPA and Zn^2+^. Light microscope images of ZnIPA show cubically shaped crystals in the range 50–100 µm (Supplementary Figure S3). Supplementary Figure S4 reported the steady-state photoluminescence (PL) and photoexcitation spectra of the ZnIPA powder, which was also consistent with the literature (Yang and Yan, 2016). Upon excitation at 300 nm, the PL spectra of ZnIPA exhibited several emission peaks at 366, 464, and 484 nm. As the excitation wavelength is swept from 250 to 350 nm, the UV emission peak showed a bathochromic shift from 366 to 392 nm (Supplementary Figure S5), indicating that there is some variance in excited-state distributions created with different excitation wavelengths. We will focus on excitation at 300 nm, as it provided a strong absorbance and was sufficiently separated from the 366 nm emission to be effectively suppressed by filters. The emission spectrum after steady-state excitation at 300 nm (52.6 mW cm^−2^) was shown in Figure 2C. The pronounced low energy peaks at 464 and 484 nm account for a significant fraction of the emission. We turned to establishing that these peaks were indeed those corresponding to the RTP, as observed in the times-series photographs in Figure 2B. Time-resolved PL measurement monitoring at 400 nm revealed that the lifetime was 1.3 ns (Supplementary Figure S6. The pulse length of 365 nm LED is 912.6 ps). Therefore, the emission at 400 nm and lower could be assigned to fluorescence (perhaps with some effects of intermolecular interactions such as π−π and Zn^2+^−π interactions in the MOF leading to not entirely monoexponential decay behavior). Utilizing a time-gating delayed detector mode, wherein emission was only recorded 0.1 ms–10 ms after an excitation pulse, the RTP spectra of ZnIPA was detected (Supplementary Figure S7, and the purple curve in Figure 2C). This gated spectrum confirms that the longer-wavelength emission in steady-state PL spectra is RTP. Now we can focus on what fraction of the emission in steady state came from singlet emission and RTP by integrating appropriate portions of the emission spectrum.

Photoluminescence quantum yield is one of the key figures-of-merit for luminescent materials and expresses the probability of photon emission per absorbed photon. We measured the absolute quantum yield of steady-state PL (QY_SS_) by using the de Mello method with an integrating sphere (deMello et al., 1997). ZnIPA exhibited 12.1% of QY_SS_ upon excitation with a 300 nm LED at a power density of 52.6 mW cm^−2^. This represented the total probability that a photon (either fluorescence or RTP) was emitted after a photon was absorbed. The probability of RTP (or fluorescence) was then just this total QY_SS_ multiplied by a factor that corresponds to the fraction of the RTP (or fluorescence in the total emission). Thus, the quantum yield of the RTP, QY_RTP_, was calculated from the ratio of the number of photons coming from RTP *versus* the number of photons coming in the total steady state PL:

$${QY}_{RTP}=\left(\frac{\int_{400}^{700}I_{RTP}\left(\lambda \right)d_{\lambda }}{\int_{320}^{700}I_{ss}\left(\lambda \right)d_{\lambda }}\right){QY}_{SS}$$ (1)

*I*_RTP_ and *I*_SS_ denote the emission intensities of RTP and steady state, respectively. With the small deviations between the blue and purple curves this method not perfect, but sufficient to estimate QY_RTP_ (to within a 10% error) in a very simple and convenient fashion (Figure 2C). For the example data above, we found that the QY_RTP_ was 3.6% under the excitation power of 52.6 mW cm^−2^. This meant that the quantum yield of the fluorescence was 8.5% and equivalently that 30% of the total emission came from RTP. Then we turned to examine how the steady state spectrum and fraction of the total emission coming from RTP changed with excitation power density.

### Effect of triplet-triplet annihilation for QY_RTP_

The crystal model based on the PXRD analysis indicated that the closest center-to-center distance between two IPA molecules was ∼4.8 Å in ZnIPA (Supplementary Figure S1), a distance consistent with triplet diffusion by intermolecular Dexter energy transfer (Meinardi et al., 2019). In addition, ZnIPA shows a triplet lifetime approaching hundreds of milliseconds at room temperature, which allows triplet excitons to migrate before spontaneous decay. This provides an opportunity for exciton collision during their diffusion process. Consequently, in addition to the monomolecular decay process, a bimolecular decay channel should be involved in the rising edge for triplet excitons (*T*). Through introducing model of TAA, the rate equation that described the rising edge at a constant excitation can be expressed as:

$$\frac{\partial T}{\partial t}=G-k_{rad}T-{\;\gamma }_{TTA\;}T^{2}$$ (2)

where *G* is the generation rate of the excitons at a certain excitation intensity, while *k*_rad_ and *γ*_TTA_ are the radiative decay rate and the second-order rate constant, respectively. The initial value condition is chosen to be *T* (t = 0) = 0, which can be solved as:

$$T=\frac{k_{rad}\;\pm \;\sqrt{k_{rad}^{2}-4\gamma_{TTA\;}G}}{-2\gamma_{TTA}}$$ (3)

By multiplying this equation with *k*_rad_ and performing the following transformations:

$$\frac{k_{rad}^{2}}{\gamma_{TTA}}\to \;C,\;k_{rad}T\to \;\tilde{T}$$ (4)

the resolving equation becomes (only a positive solution is considered):

$$\tilde{T}=\frac{C+\sqrt{C^{2}-4CG}}{2}$$ (5)

**FIGURE 3:**
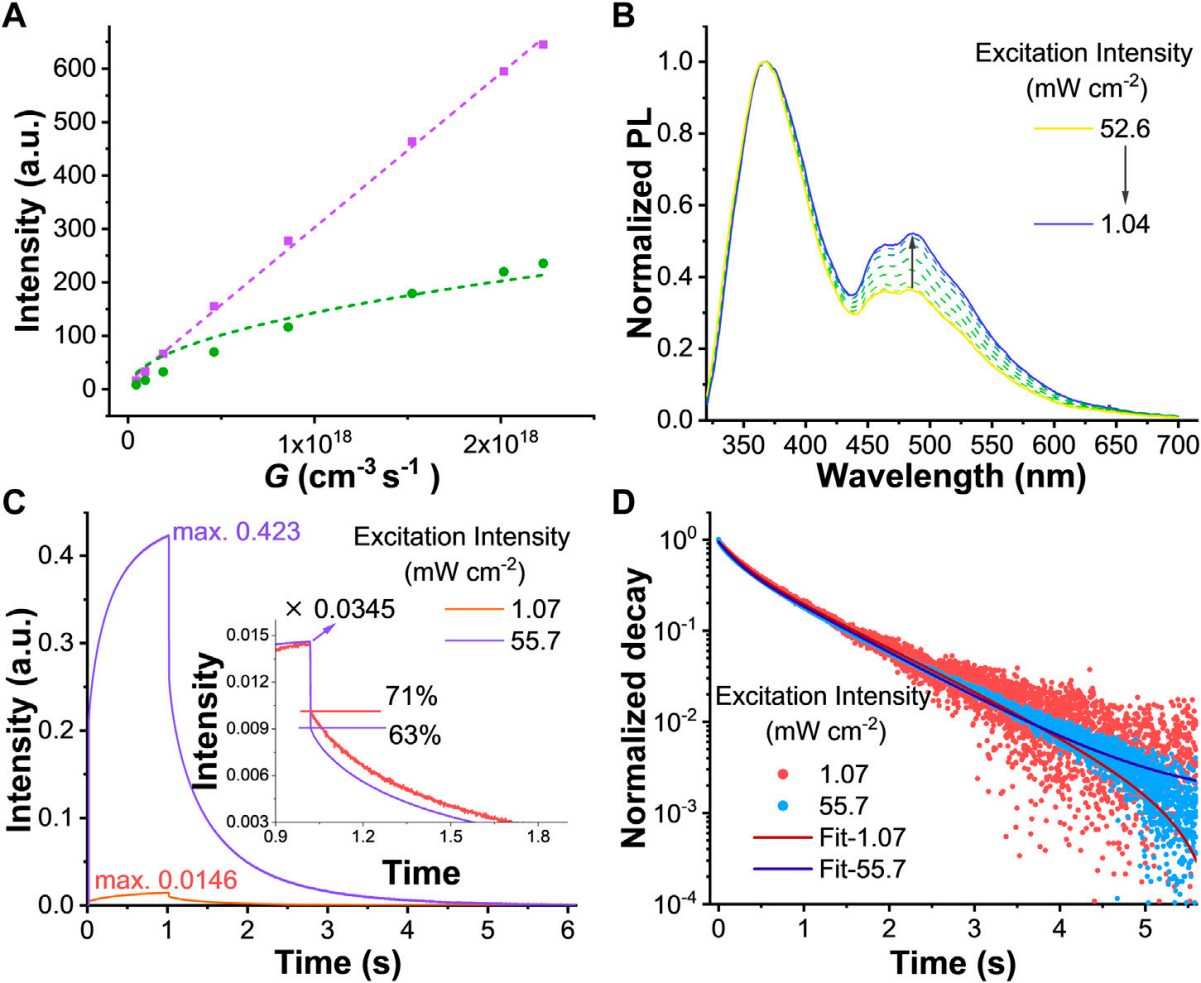
**(A)** Power dependent PL intensities are monitored at 366 (purple square) and 484 (green triangle) nm. Purple dash line is obtained from a linear fit while green dash line results from a global fit using Eq. 5 **(B)** Normalized PL spectra of ZnIPA under different excitation power **(C)** Time-resolved PL spectra of ZnIPA under different excitation power. The insert is a zoomed spectra with time range from 0.9 to 1.9 s. A 500 nm long pass filter is setup before the detector to remove the most parts of fluorescence **(D)** Normalized decay spectra of ZnIPA, the fitting lines are obtained using a biexponential method. All the samples are under excitation of 300 nm LED.

Eq. 5 presents a description of the emission intensity as a function of the excitation power: the RTP increases nonlinearly with excitation power density if the second order annihilation rate *k*_TTA_ plays a role. This peculiar triplet dynamic can be evidenced by means of power-dependent PL intensity. Indeed, as shown in Figure 3A, the fluorescence at 366 nm increased linearly (purple) because of much less influence of exciton-exciton annihilation, while the emission at 484 nm (green) exhibited a sublinear dependence on the excitation power. The fitting (green line) of RTP using Eq. 5 gives a ratio $k_{rad}^{2}$ /*γ*_TTA_ = 7.95 × 10^10^ cm^−2^ s^−1^. Taking *k*_rad_ = (779 ms)^−1^, as obtained later, we calculated a second order constant *γ*_TTA_ = 2.06 × 10^−11^ cm^3^ s^−1^. Additionally, Figure 3B presented the changing steady-state emission spectra as a function of excitation power density coming from the 300 nm LED. As the excitation power density decreased, the intensities of RTP shoulder were getting stronger compared to the fluorescence. It was worth pointing out that both the QY_SS_ and QY_RTP_ values increased when lowering the excitation power density. We calculated the QY_SS_ under different excitation intensities by using a relative quantum yield method (see in the Supplementary Material). The QY_SS_ gradually increased as the power density was reduced, reaching a maximum of 17.5% at an excitation intensity of 1.04 mW cm^−2^. The calculated QY_RTP_ also steadily raised to 6.4% at low power (Supplementary Table S1). Meanwhile, we noted that the low RTP efficiency resulted from the enhancement of TTA at high power density, which agrees with the triplet dynamic model.

The steady state data already demonstrated that TTA reduced the RTP efficiency at high excitation power densities. Another common method of examining TTA is to observe the power dependence of the triplet lifetime. As the triplet density increases with high-power excitation, TTA will cause a shortening of RTP lifetime. Time-resolved PL spectra showed a 29-times improvement at the maximum emission intensity (Figure 3C) while the excitation intensity increased from 1.07 to 55.7 mW cm^−2^ (52-fold). In addition, a fast drop was observed after the LED was turned off due to the decay of residual fluorescence. By adjusting these two spectra to the same level, the ratio of RTP exhibited an obvious decease from 71% to 63% (fast decay increased from 29% to 37%) at high power (see inset of Figure 3C). Such behavior agreed with the model of TTA effect. Unexpectedly, as shown in Figure 3D, the change in the long tail was minimal. The lifetimes of RTP remained almost unaffected as a function of excitation power density (Supplementary Table S2). We suggested the origin of this disagreement with our hypothesis, could likely be due to the confinement effect in MOF crystals. For instance, when triplets were generated in a physically confined environment (such as a single cubic-like grain), they will undergo an ultra-fast decay until there was only isolated triplets per grain left. Afterwards, the remaining isolated triplet transited with the constant rate presented by the long tail.

### Extended observation in ZnTPA

**FIGURE 4:**
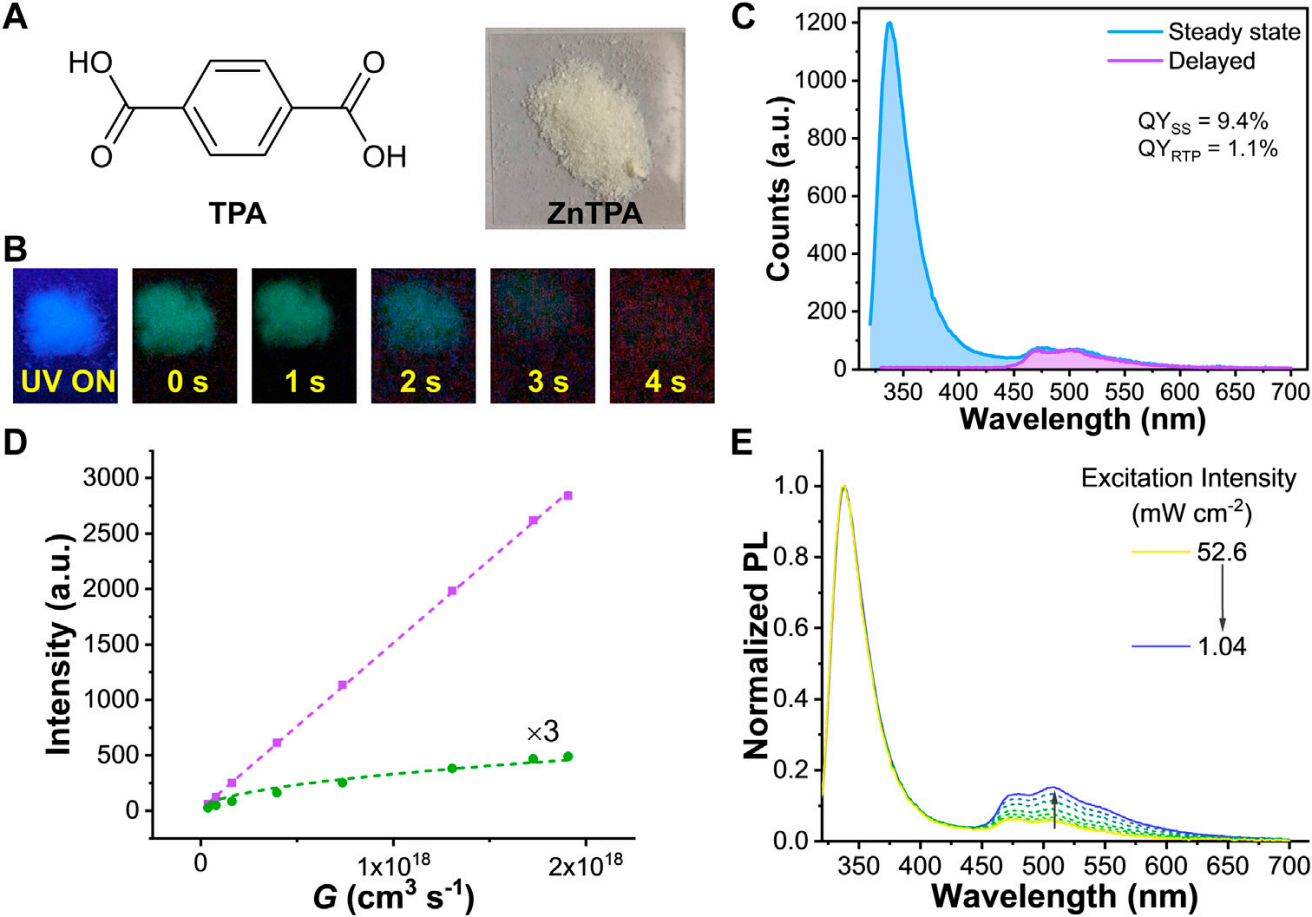
**(A)** Chemical structure of terephthalic acid (TPA) and photograph of ZnTPA MOF **(B)** Photographs of ZnTPA under and after excitation of UV (365 nm) light **(C)** Steady-state PL and delayed RTP spectra of ZnTPA. The blue- and purple-colored fillings represent the integrating areas of steady-state PL and delayed RTP, respectively. For steady-state PL and QY_SS_, the excitation power is 52.6 mW cm^−2^ **(D)** Power dependent PL intensities are monitored at 338 (purple square) and 509 (green rhombus) nm. Purple dash line is obtained from a linear fit while green dash line results from a global fit using Eq. 5 **(E)** Normalized PL spectra of ZnTPA under different excitation power. All the PL spectra were carried out using excitation at 300 nm.

The effect of TTA in organic RTP was not limited to the ZnIPA MOF. We also tested the terephthalic acid-based zinc MOF (ZnTPA). Same as ZnIPA, PXRD, FTIR, and optical microscope measurements were carried out to verify the formation of ZnTPA (Supplementary Figures S8-S10). Under UV illumination, the white powder of ZnTPA showed cyan-blue emission followed by a green afterglow (Figures 4A,B). The steady-state photoexcitation and photoluminescence spectra revealed that the ZnTPA could be excited under UV light and produced emission with peaks at 338, 480, and 509 nm (Supplementary Figure S11). The peaks of phosphorescence located at 473 and 503 nm in ZnTPA, suggesting the shoulder at long wavelength region was RTP emission (Supplementary Figure S12). Accordingly, the QY_SS_ and QY_RTP_ were calculated to 9.4% and 1.1%, respectively (Figure 4C). The closest center-to-center distance between two TPA molecules was ∼5.0 Å (Supplementary Figure S10), implying triplet diffusion can also happen in ZnTPA. By varying the excitation intensity, the linear singlet emission and multiexponential RTP emission were observed (Figure 4D) and the ratio of RTP changed in the steady-state PL spectra (Figure 4E). In addition, the total QY_SS_ and the QY_RTP_ exhibited a rising trend as a function of decreasing power density, finally reaching 11.1% and 2.7%, respectively, at 1.04 mW cm^−2^ (Supplementary Table S3). Time-resolved PL spectra revealed that the long tail of RTP remained stable while the fraction of fluorescence increased at the high power (Supplementary Figure S13 and Supplementary Table S4). These results using ZnTPA were similar with ZnIPA, confirming the generality of the effect of TTA in RTP materials.

## Conclusion

In summary, we demonstrated that the effect of TTA can suppress the quantum yield of RTP in MOFs. The long triplet lifetime and good triplet diffusion mean that RTP MOFs are susceptible to TTA. TTA reduces the efficiency of triplet emission even at excitation power densities in the range of mW cm^−2^ (due to the very long lifetime of the triplet states in the material). This means that the ratio of singlet to triplet emission is dependent on the excitation power density, and the RTP becomes much weaker relative to the singlet emission as the excitation power density is increased. In Zn IPA, the ratio of RTP to fluorescence dramatically decreased from 0.58 to 0.42 with the excitation power changed from 1.04–52.6 mW cm^−2^. The change of fraction clearly indicated that TTA affected the RTP efficiency owing to the fluorescence was linearly dependent on the excitation intensity, while RTP showed a quadratic relationship. This means that use of such materials for a ratiometric sensor will be challenging, as the ratio of singlet to triplet emission will depend on the excitation power density, as well as the desired external factor that is wished to be sensed.

However, when the time-resolved emission was investigated, only a greater fraction of fast decay was observed for increasing power whereas the lifetime of the long tail was unchanged. This may be because of the special confinement of triplets in MOF crystals. This behavior is fortunate for lifetime sensing, as quenching of the long tail by environmental factors could be used for a sensor that is not affected by the excitation power density. This study of triplet dynamics in RTP MOFs offers a deepened fundamental understanding of the materials and a more nuanced insight into their development for sensing applications.

## Experimental section

Synthesis of ZnIPA MOFs: 88 mg of IPA (99%, Sigma-Aldrich), 82 mg of Hmim (99%, Sigma-Aldrich), 150 mg of Zn(NO_3_)_2_⋅6H_2_O (99%, Alfa Aesar), and 8 ml of water are mixed in a G30 vial (volume of 25 ml). The mixture is sonicated to disperse all the components, and the vial is placed inside the microwave reactor (Monowave 400, Anton Paar) at 180°C for 3 h. After the mixture cools to room temperature, ZnIPA powder is washed with acetone in order to substitute the residual water in the pores. Finally, ZnIPA powder is activated in a vacuum oven by keeping at 100°C for 24 h.

Synthesis of ZnTPA MOFs: 88 mg of TPA (98%, Merck KGaA), 41 mg of Hmim, 150 mg of Zn(NO_3_)_2_⋅6H_2_O, and 8 ml of water are mixed in a G30 vial (volume of 25 ml). The mixture is sonicated to disperse all the components, and the vial is placed inside the microwave reactor at 180°C for 50 min. After the synthesis, ZnTPA powder is activated by the same method as ZnIPA.

## Data Availability

The original contributions presented in the study are included in the article/Supplementary Material, further inquiries can be directed to the corresponding author.
