# Supplementary material for: Limitation of room temperature phosphorescence efficiency in metal organic frameworks due to triplet-triplet annihilation
Source: Front Chem. 2022 Oct 31;10:1010857. doi: 10.3389/fchem.2022.1010857 (PMC9659923; doi:10.3389/fchem.2022.1010857)
Supplement: Supplementary file 1 [file DataSheet1.docx]

Supplementary Material

# Materials and Characterization

*Materials.* All regents and solvents were used as received otherwise indicated. Terephthalic acid (TPA) was purchased from Merck KGaA. Isophthalic acid (IPA) and 2-methylimidazole (Hmim, 99%) were purchased from Sigma-Aldrich GmBH. Zinc nitrate hexahydrate (Zn(NO_3_)_2_⋅6H_2_O, 99%, Alfa Aesar) was purchased form ThermoFisher (Kandel) GmbH.

*Characterization.* Powder X-ray diffraction patterns were obtained using a D2 Phaser diffractometer (Bruker) with CuK*α* radiation. Steady-state photoluminescence and photoexcitation spectra were carried out on Edinburgh FS5 spectrofluorometer with an internal Xe lamp excitation source. Power-dependent photoluminescence spectra were recorded using a spectrometer (AvaSpec-2048x64TEC, Avantes) system attached to a UV LED (*λ* = 300 nm) excitation source. Phosphorescence spectra and long-lived lifetime were also measured on Edinburgh FS5 spectrofluorometer using an internal microsecond flashlamp (μF lamp) as illumination source. Power-dependent emission decay were performed on a USB spectrometer with an amplified Si detector (PDA100A2) upon excitation of a UV LED (*λ* = 300 nm), a 500 nm long pass filter was setup before the detector. Fluorescence decay was recorded on Edinburgh FS5 spectrofluorometer under excitation of an external LED (365 nm). Addition to an integrating sphere (15-cm diameter, Labsphere), the absolute PL quantum yields were also performed on the AvaSpec-2048x64TEC spectrometer and using 300 nm UV LED as excitation source. Fourier-transform infrared (FTIR) spectra were carried out on VERTEX 70 FT-IR spectrometer (Bruker). Optical microscope images were captured by Keyence VH-Z500R. All those characterizations were carried out under ambient condition.

**2** **Supplementary Figures and Tables**


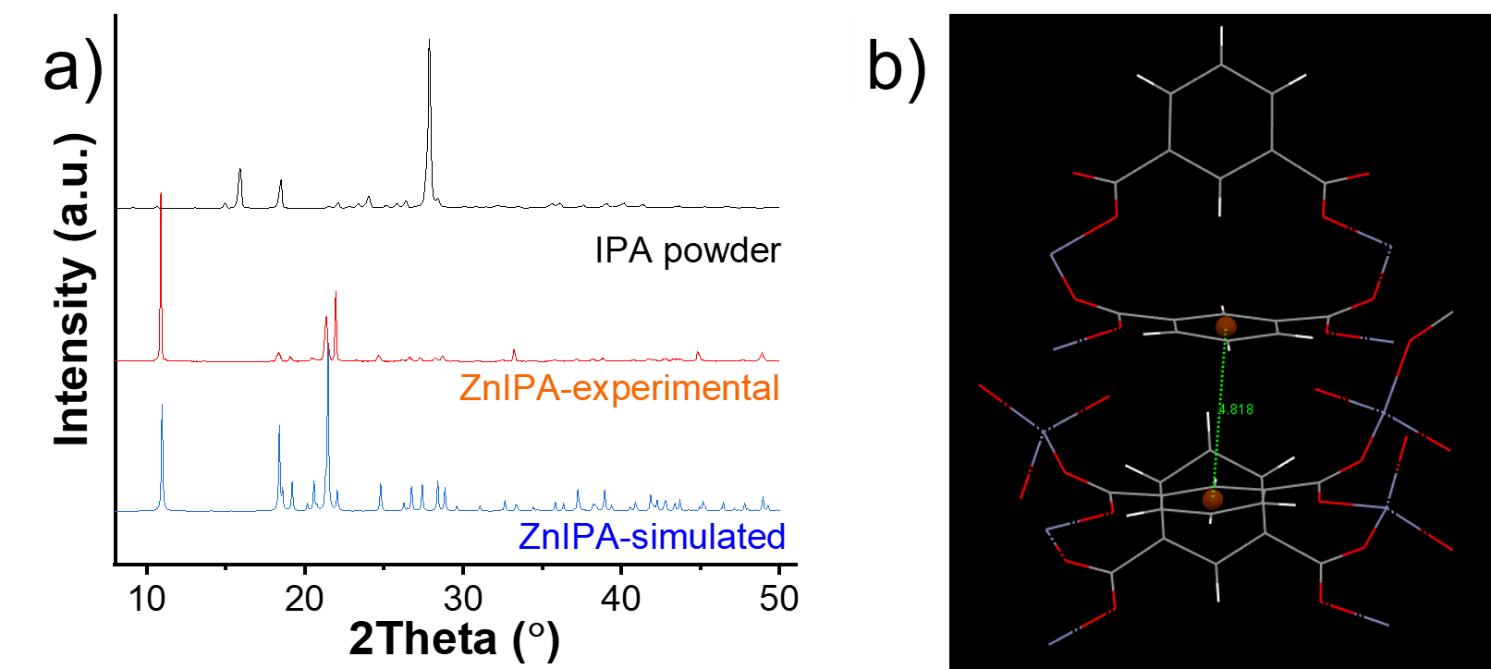


**Supplementary Figure 1.** (a and b) PXRD pattern of IPA and ZnIPA MOF with corresponding simulated patterns made from their crystal structures. Structure comes from .cif file: https://www.ccdc.cam.ac.uk/structures/Search?Ccdcid=1432069&DatabaseToSearch=Published.


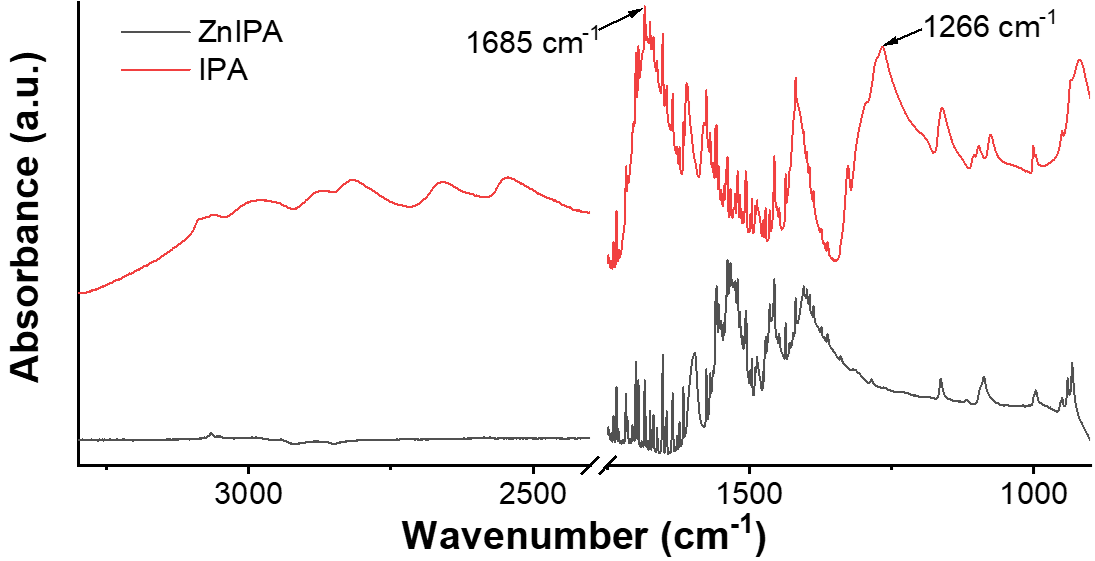


**Supplementary Figure 2.** FTIR spectra of IPA and ZnIPA powder.


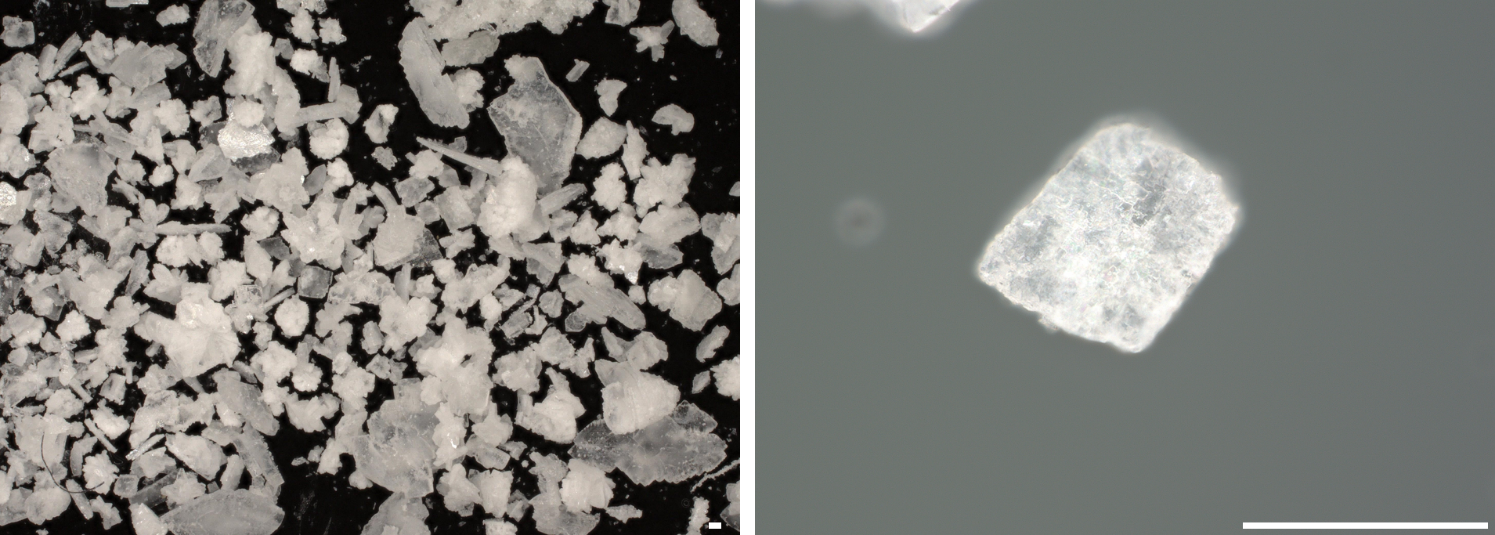


**Supplementary Figure 3.** Microscopic images of ZnIPA. Scale bar = 100 μm.

**
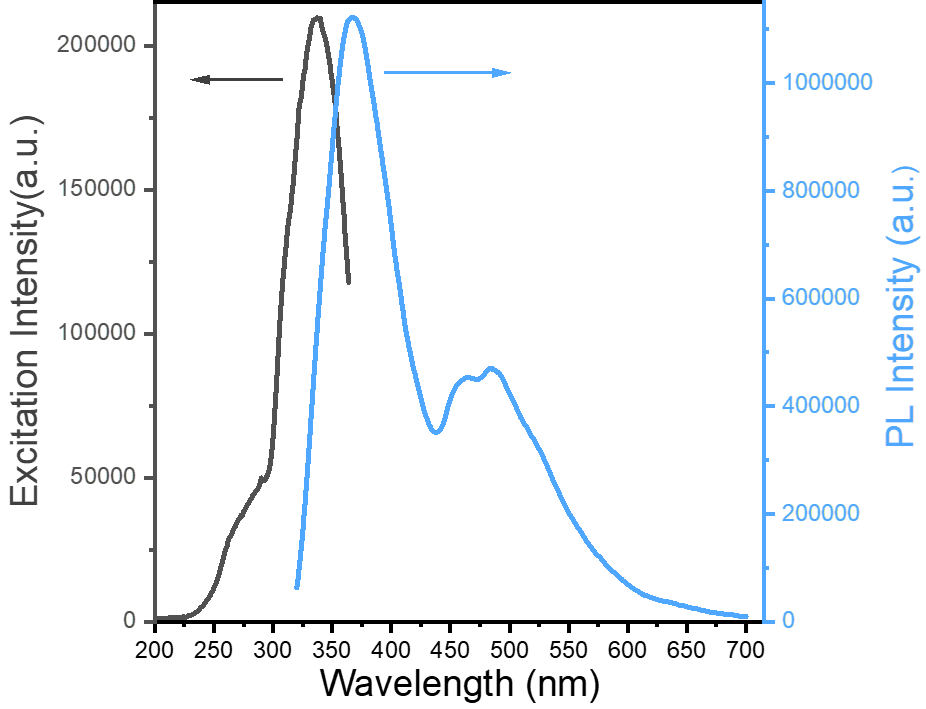
**

**Supplementary Figure 4.** Photoexcitation and photoluminescence (PL) spectra of ZnIPA powder. *λ*_ex_ = 300 nm.

**
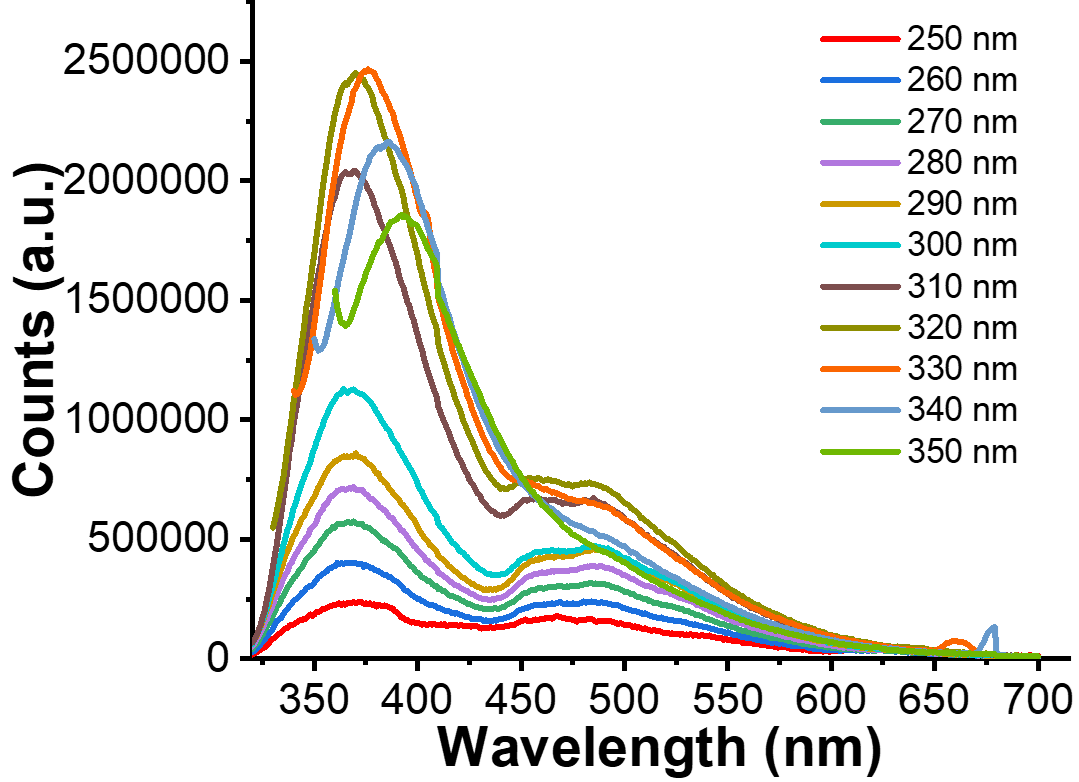
**

**Supplementary Figure 5.** PL spectra of ZnIPA powder as a function of different excitation wavelength.

**
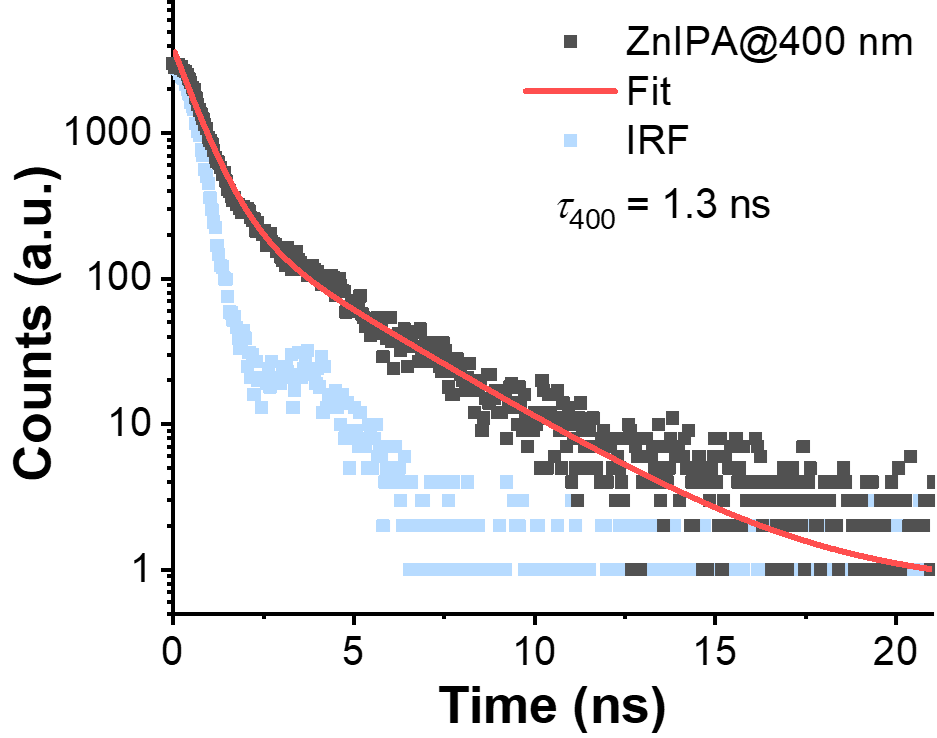
**

**Supplementary Figure 6.** Fluorescence decay of ZnIPA powder. The pulse length of 365 nm LED is 912.6 ps. *λ*_ex_ = 365 nm.

**
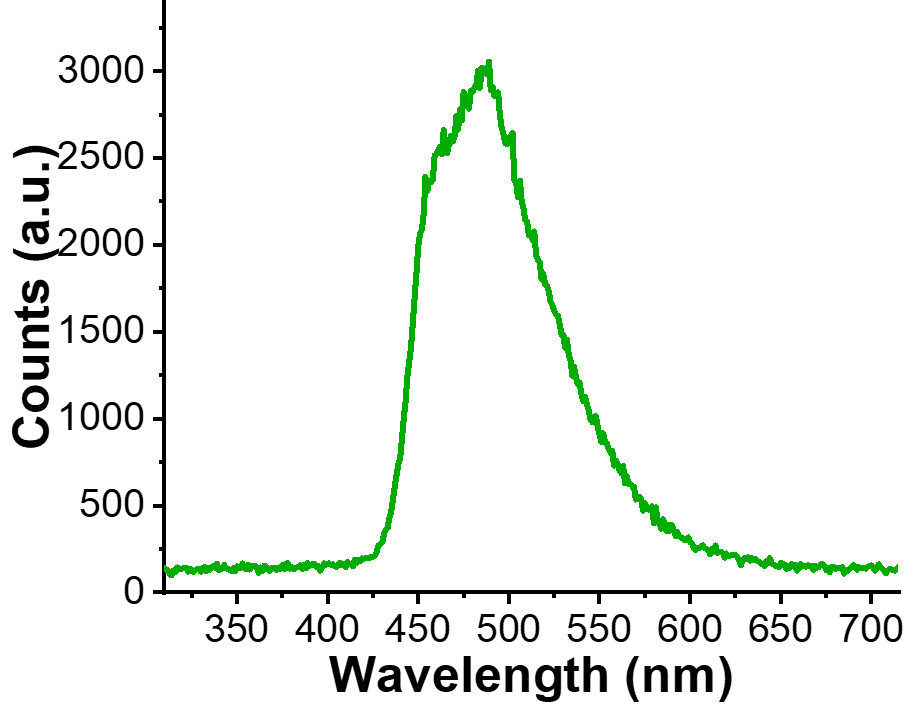
**

**Supplementary Figure 7.** Phosphorescence spectra of ZnIPA powder. Gating from 0.1 ms to 10 ms. *λ*_ex_ = 300 nm.

The relative quantum yield values QY_unk_ are obtained according to the following relation:

$\mathbf{QY}_{\mathbf{unk}}\mathbf{=}\mathbf{QY}_{\mathbf{ref}}\left( \frac{\mathbf{E}_{\mathbf{ref}}}{\mathbf{E}_{\mathbf{unk}}} \right)\left( \frac{\mathbf{A}_{\mathbf{ref}}}{\mathbf{A}_{\mathbf{unk}}} \right)\left( \frac{\mathbf{I}_{\mathbf{unk}}}{\mathbf{I}_{\mathbf{ref}}} \right)\left( \frac{\mathbf{n}_{\mathbf{unk}}}{\mathbf{n}_{\mathbf{ref}}} \right)^{\mathbf{2}}$

where QY is the quantum yield, *E* is the excitation intensity, *A* is the fraction of photons absorbed at the excitation wavelength, *I* is the integrating photoluminescence intensity, and *n* is the medium refractive index. The subscripts *unk* and *ref* denote the parameters relative to the unknown and reference standard systems (QY_ref_ = QY_SS_ = 12.1%).

**Table S1.** QY_SS_ and QY_RTP_ of ZnIPA under different power intensity.

| **Excitation**  **(mW cm^-2^)** | **QY_SS_**  **(%)** | **QY_RTP_**  **(%)** |
| --- | --- | --- |
| 52.6 | 12.1 | 3.6 |
| 47.6 | 12.4 | 3.8 |
| 36.0 | 13.0 | 4.0 |
| 20.3 | 14.2 | 4.6 |
| 10.9 | 15.3 | 5.2 |
| 4.46 | 16.6 | 5.9 |
| 2.18 | 17.3 | 6.2 |
| 1.04 | 17.5 | 6.4 |

**Table S2.** RTP lifetimes of ZnIPA under different power intensity.

| **Excitation**  **(mW cm^-2^)** | ***τ*_1_**  **(ms)** | ***τ*_2_**  **(ms)** | ***τ*_ave_**  **(ms)** |
| --- | --- | --- | --- |
| 1.74 | 250 | 934 | 822 |
| 6.44 | 228 | 897 | 799 |
| 16.6 | 209 | 877 | 789 |
| 55.7 | 198 | 865 | 779 |

**
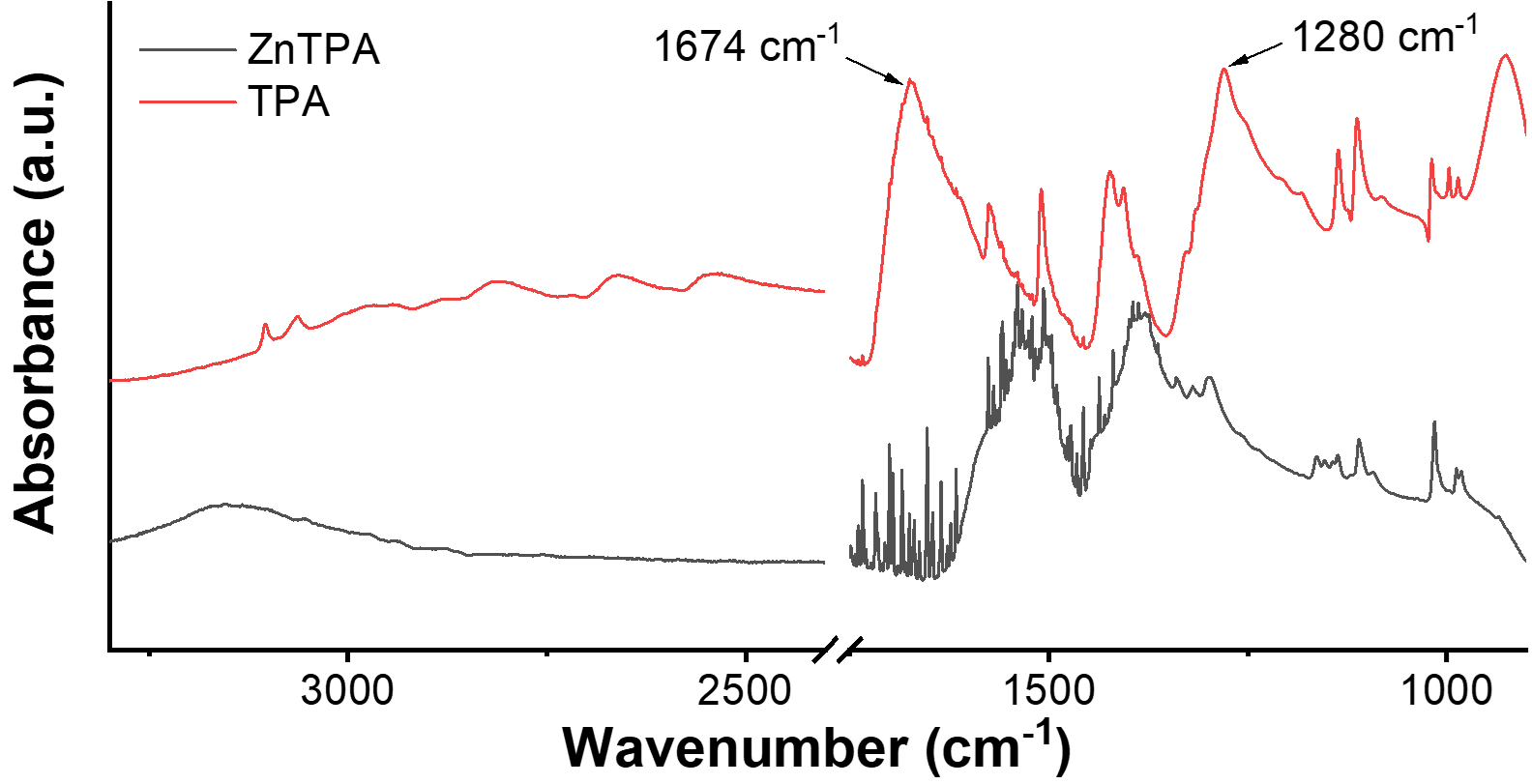
**

**Supplementary Figure 8.** FTIR spectra of TPA and ZnTPA powder.

**
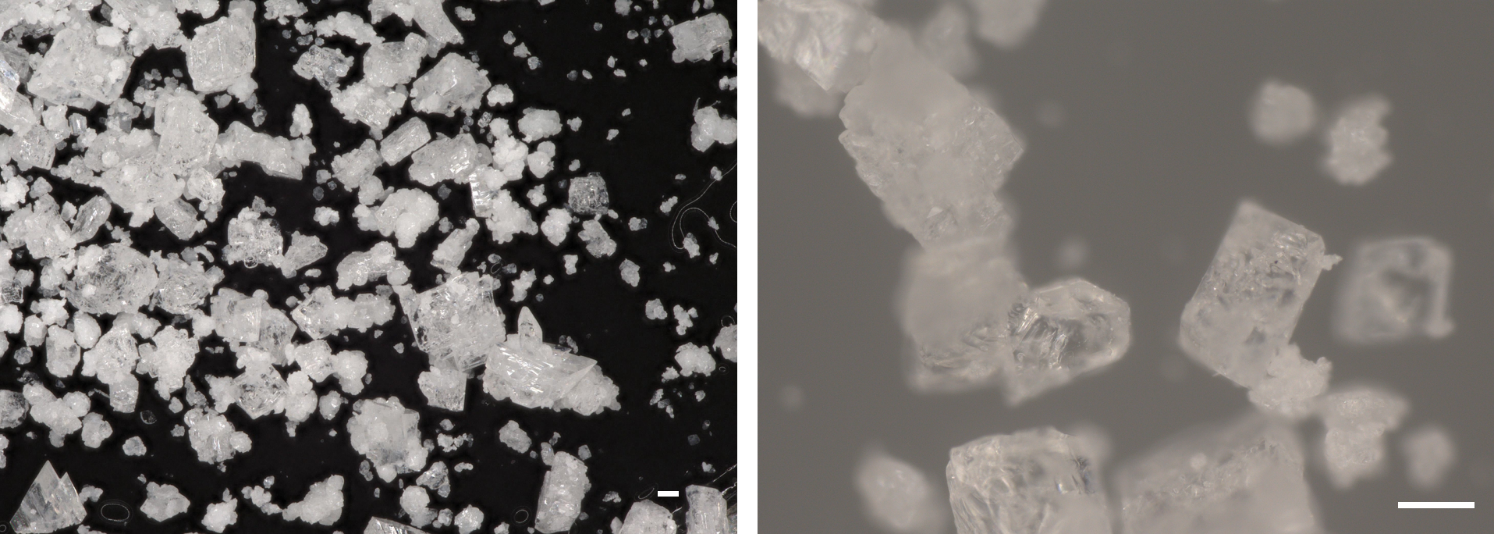
**

**Supplementary Figure 9.** Microscopic images of ZnTPA. Scale bar = 100 μm.

**
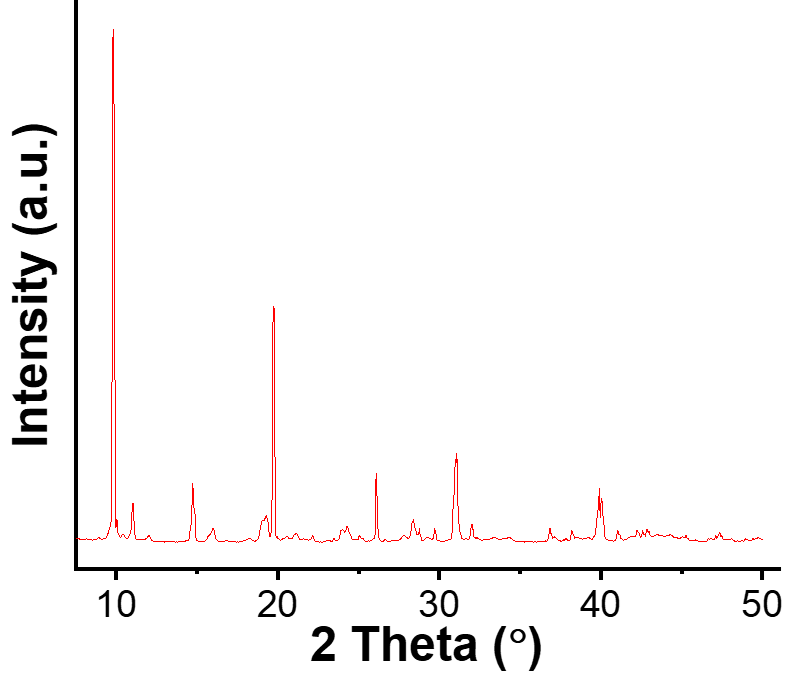
**

**Supplementary Figure 10.** PXRD pattern of ZnTPA MOF.

**
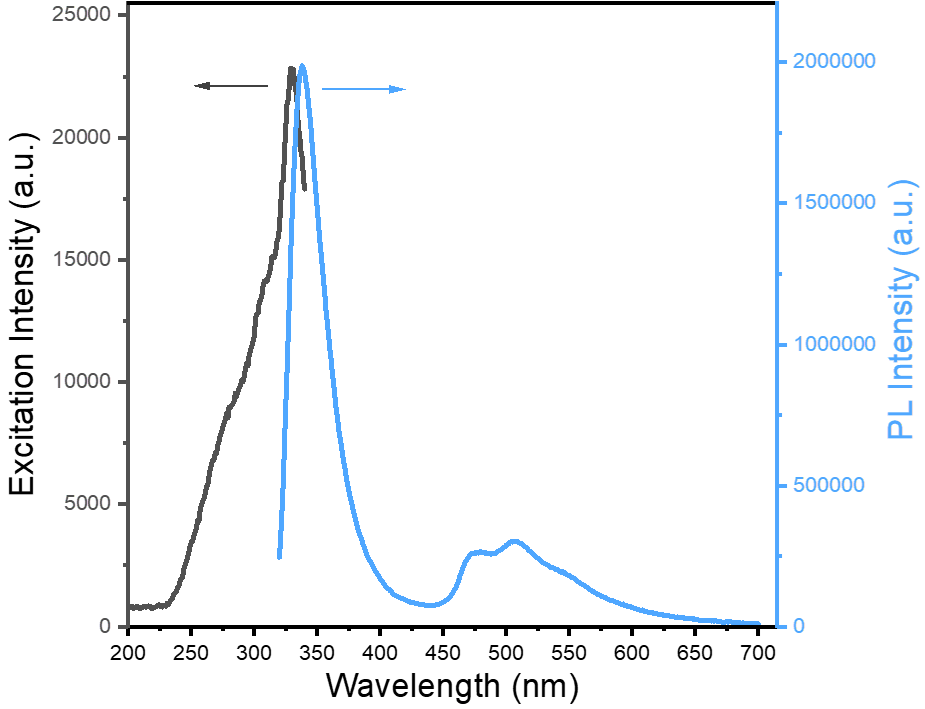
**

**Supplementary Figure 11.** Photoexcitation and P) spectra of ZnTPA powder. *λ*_ex_ = 300 nm.

**
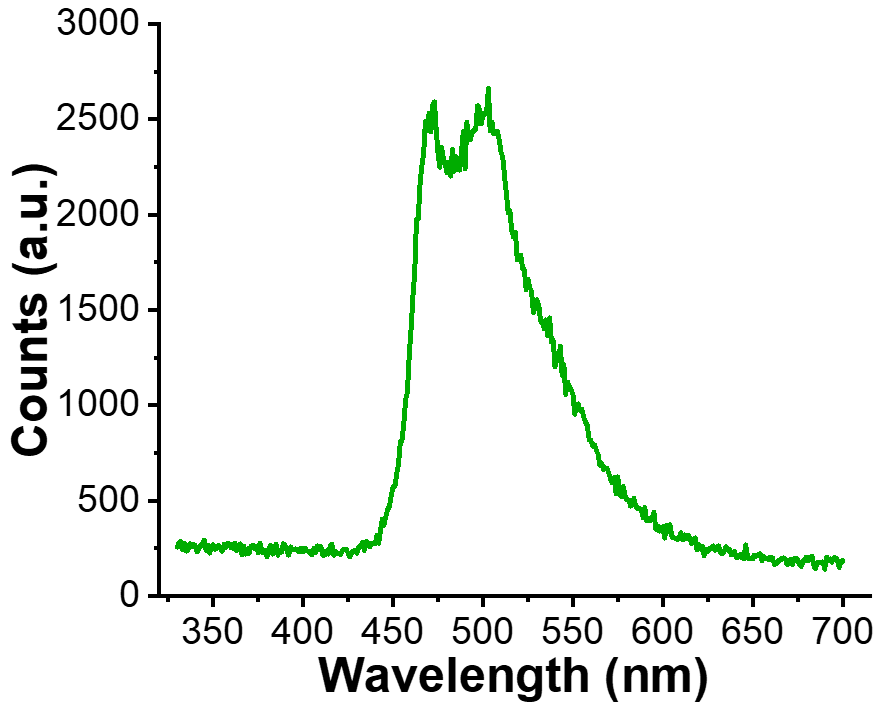
**

**Supplementary Figure 12.** Phosphorescence spectra of ZnTPA powder. Gating from 0.1 ms to 10 ms. *λ*_ex_ = 300 nm.

**
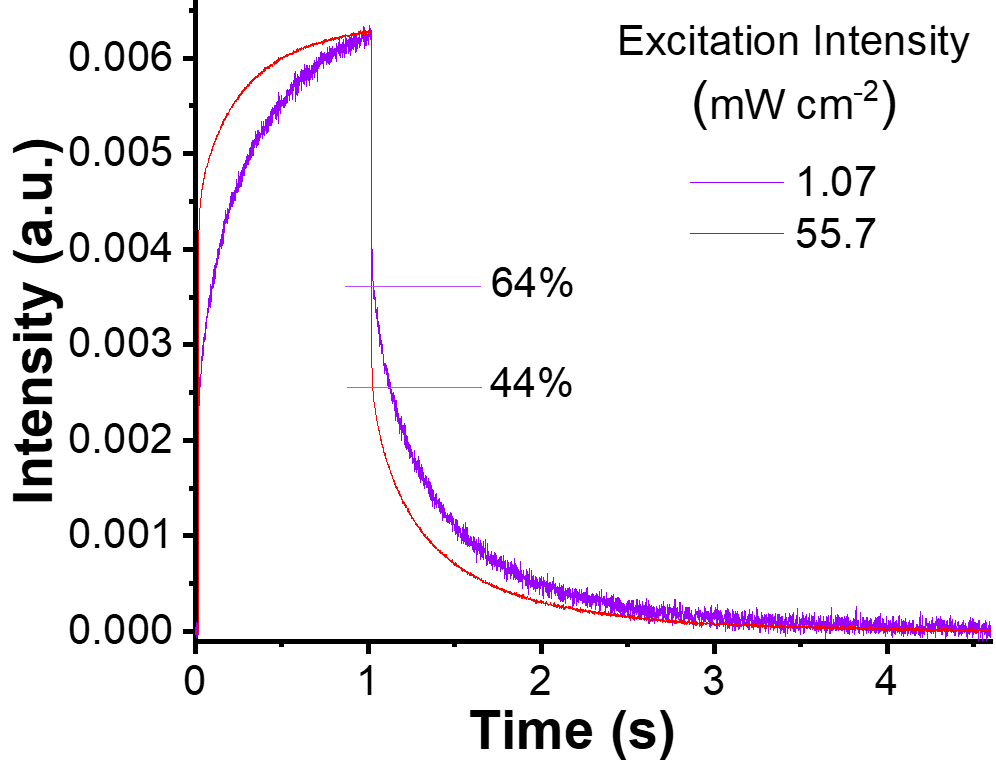
**

**Supplementary Figure 13.** Time-resolved PL spectra of ZnTPA as a function of different excitation power at 300 nm. A 500 nm long pass filter is setup before the detector to remove the most parts of fluorescence.

The relative quantum yield values of ZnTPA are calculated using the same method as ZnIPA, and QY_ref_ = QY_SS_ = 9.4%.

**Table S3.** QY_SS_ and QY_RTP_ of ZnTPA under different power intensity.

| **Excitation**  **(mW cm^-2^)** | **QY_SS_**  **(%)** | **QY_RTP_**  **(%)** |
| --- | --- | --- |
| 52.6 | 9.4 | 1.1 |
| 47.6 | 9.6 | 1.2 |
| 36.0 | 9.7 | 1.4 |
| 20.3 | 10.0 | 1.5 |
| 10.9 | 10.2 | 1.8 |
| 4.46 | 10.6 | 2.1 |
| 2.18 | 10.9 | 2.4 |
| 1.04 | 11.1 | 2.7 |

**Table S4.** RTP lifetimes of ZnTPA under different power intensity.

| **Excitation**  **(mW cm^-2^)** | ***τ*_1_**  **(ms)** | ***τ*_2_**  **(ms)** | ***τ*_ave_**  **(ms)** |
| --- | --- | --- | --- |
| 1.74 | 195 | 791 | 660 |
| 6.44 | 160 | 694 | 602 |
| 16.6 | 144 | 665 | 588 |
| 55.7 | 139 | 664 | 586 |
